# Adaptation and Validation of a Spanish Writing Self-Efficacy Scale in Quechua-Speaking Peruvian Basic Education Students

**DOI:** 10.3390/bs15101418

**Published:** 2025-10-18

**Authors:** Moises Curo-Huacani, Liset Z. Sairitupa-Sanchez, Gutember Peralta-Eugenio, Mardel Morales-García, Róbert-János Ilyés, Wilter C. Morales-García

**Affiliations:** 1 Unidad de Educación, Escuela de Posgrado, Universidad Peruana Unión, Ñaña, Lurigancho-Chosica, Lima 15464, Peru; 2 Unidad de Psicología, Escuela de Posgrado, Universidad Peruana Unión, Ñaña, Lurigancho-Chosica, Lima 15464, Peru; 3 Facultad de Ciencias de la Salud, Escuela de Psicología, Universidad Cesar Vallejo, Chimbote 02801, Peru; 4 Unidad de Salud, Escuela de Posgrado, Universidad Peruana Unión, Ñaña, Lurigancho-Chosica, Lima 15464, Peru; 5 Ecumene Doctoral School, Faculty of Reformed Theology and Music, Babeș–Bolyai University, 400084 Cluj-Napoca, Romania; robert.ilyes@uadventus.ro; 6 Dirección General de Investigación, Universidad Peruana Unión, Ñaña, Lurigancho-Chosica, Lima 15464, Peru

**Keywords:** writing self-efficacy, scale validation, Quechua-speaking students, confirmatory factor analysis, educational psychometrics

## Abstract

*Background:* Writing self-efficacy is a central construct in educational research, grounded in Bandura’s social cognitive theory. However, most available instruments have been developed in Western and urban contexts, which limits their applicability to indigenous bilingual populations, such as Quechua-speaking students in Peru. The absence of validated scales in these contexts hinders the accurate assessment of writing self-efficacy and the implementation of educational strategies tailored to their linguistic and cultural needs. *Objective:* This study aimed to adapt and validate the Writing Self-Efficacy Scale (QEWSE) for Quechua-speaking students in basic education in Peru, ensuring its structural validity and reliability. *Methods:* An instrumental study was conducted with a sample of 265 secondary school students (50.6% female, 49.4% male), using convenience sampling. Confirmatory factor analysis (CFA) was applied to evaluate the structure of the instrument. Reliability was assessed using Cronbach’s alpha and McDonald’s omega coefficients. *Results:* The four first-order factor model (Ideation, Skills, Usage, and Self-Regulation) showed adequate fit indices (CFI = 0.92; TLI = 0.91; RMSEA = 0.05 [90% CI: 0.05–0.06]; SRMR = 0.05). However, the high inter-factor correlations (≥0.85) suggest the relevance of a second-order model, which demonstrated a reasonable fit (CFI = 0.92; TLI = 0.91; RMSEA = 0.06; SRMR = 0.05), supporting the interpretation of writing self-efficacy as a global construct. The scale showed high reliability across all factors (α and ω ranged from 0.74 to 0.90). *Conclusions:* The QEWS-S demonstrates strong psychometric properties for assessing writing self-efficacy among Quechua-speaking students. The hierarchical second-order model offers a more accurate theoretical and empirical representation, allowing for the reporting of an overall self-efficacy score while also providing specific scores for each dimension. These results support its use in bilingual and culturally diverse contexts and lay the groundwork for future research aimed at further examining discriminant validity and developing pedagogical interventions focused on strengthening students’ confidence and writing skills.

## 1. Introduction

Writing self-efficacy is a fundamental construct for understanding why, how, and to what extent students engage in, regulate, and improve their writing performance in formal learning contexts. This construct integrates beliefs about one’s own abilities with motivational and self-regulatory processes, which are closely linked to academic achievement and persistence when facing complex tasks inherent to the development of advanced literacy (Bandura, 1997; Pajares, 2003). From the perspective of social cognitive theory, self-efficacy is defined as the judgments individuals make about their ability to organize and execute the actions required in specific situations. In the case of writing, these judgments manifest in planning, drafting, revising, and editing texts for various purposes and genres, directly influencing goal selection, the effort invested, tolerance for frustration, and resilience in the face of errors (Bandura, 1997). Within the educational setting, writing self-efficacy holds particular relevance. When students believe in their ability to write, they tend to engage more actively and strategically in writing tasks, experience less apprehension toward writing, and achieve better performance, even when controlling for differences in aptitude and prior knowledge (Pajares, 2003; Pajares & Valiante, 1997). Furthermore, confidence in writing skills not only enhances the quality of the texts produced but also strengthens persistence when facing challenges and promotes resilience in response to negative feedback, which is essential for the continuous development of writing competence (Graham & Harris, 2018; Pajares, 2002). Writing self-efficacy is therefore considered a critical predictive variable for academic achievement. Its development depends on factors such as successful past experiences, social feedback, and observation of successful models, which help students build a positive perception of their abilities and maintain the effort needed to reach advanced levels of writing performance (Bandura, 1997; Klassen, 2002).

In basic education, these beliefs play a central role in both the academic and emotional development of students, showing consistent relationships with indicators of achievement, motivation, and perceived value. Writing self-efficacy develops and evolves throughout primary and secondary education, directly influencing both performance and intrinsic motivation for writing (Pajares, 2003; Pajares & Valiante, 1997). Several studies have demonstrated that self-efficacy not only predicts writing performance but also mediates the relationship between cognitive and motivational variables involved in text production. It plays a proximal role in the motivational architecture of the writing process during childhood and adolescence (Pajares & Valiante, 1997, 1999, 2001). Students with high levels of self-efficacy are more likely to start and complete writing tasks willingly, develop more effective strategies, and experience less anxiety and apprehension about writing compared to those with low self-efficacy (Pajares, 2003; Pajares & Valiante, 1997). These effects have been consistently observed across diverse contexts and educational levels, emphasizing the importance of addressing self-efficacy as a key objective in writing instruction (Pajares & Valiante, 1997). However, during the transition from primary to secondary education, a decline in self-efficacy levels is often observed. This decrease is attributed to the increase in academic demands and the greater complexity of writing tasks. Such a decline can be reversed through specific pedagogical interventions that foster mastery experiences, positive feedback, and opportunities for guided practice. These interventions have been shown to improve both the quality of students’ written texts and their confidence in their writing abilities (Graham & Perin, 2007; Shell et al., 1989). Research has also identified gender differences in these beliefs. Girls tend to report greater perceived value toward writing and lower apprehension toward the task, while boys may demonstrate higher confidence in specific contexts depending on the type of text and subject being evaluated. These differences are not static and tend to vary according to grade level and the cultural expectations of the educational environment (Pajares & Valiante, 1999, 2001). Finally, writing self-efficacy is closely related to reading skills. Confidence in text comprehension strengthens confidence in text production, as both processes share cognitive and linguistic components. This interrelationship suggests that comprehensive programs that simultaneously promote reading and writing can generate more sustainable improvements in students’ overall learning (Shell et al., 1989).

Despite the importance of this topic, the literature reveals significant gaps. Most writing self-efficacy scales have been developed and validated in monolingual Western contexts, primarily in English or in Spanish as a dominant language, which limits their applicability to bilingual and Indigenous populations (Gebauer et al., 2021; Ramírez Rodríguez, 2022). This methodological bias is particularly problematic in Quechua-speaking communities, where learning to write in Spanish occurs under conditions very different from those experienced by monolingual populations. In these cases, a literal translation of items is insufficient to capture the cultural and conceptual nuances that shape the experience of learning and writing in a second language. Certain terms and concepts may be unfamiliar or carry different meanings for students whose initial literacy took place primarily in Quechua before they entered a school system where Spanish is the language of instruction (Hambleton & Patsula, 1998; Muñiz et al., 2013). Therefore, the challenge lies not only in linguistic translation but also in ensuring that the items accurately represent the construct of writing self-efficacy while taking into account the perspective and lived experiences of Quechua–Spanish bilingual students. Without appropriate cultural adaptation, there is a risk of misinterpreting results, potentially attributing a supposed lack of ability to students when, in fact, the difficulties reflect the process of learning Spanish and the influence of a pedagogical context shaped by structural inequalities (Ames et al., 2011; Flores & Rosa, 2015; Hynsjö & Damon, 2015). This misinterpretation has consequences that extend beyond academic evaluation. It can negatively affect pedagogical decisions and teacher feedback, potentially perpetuating inequities and impacting students’ educational trajectories, self-esteem, and long-term social integration (UNESCO, 2022; UNESCO et al., 2015). Consequently, it is essential to develop culturally relevant instruments that recognize the linguistic and cultural strengths of these communities, avoiding decontextualized assessments and promoting equitable education (Bandura, 2006; Zimmerman, 2000).

Various instruments have been designed to assess writing self-efficacy. Among them, Pajares’ (2007) scale stands out as it evaluates both basic and advanced writing skills. Its adaptation to the Peruvian context demonstrated factorial invariance among secondary school students (León-Gutiérrez et al., 2023). More recently, Sun et al. (2022) developed an English-language scale to measure writing self-efficacy in students learning English as a second language. This instrument is based on a three-dimensional model that assesses ideation, conventions, and self-regulation, providing a comprehensive view of the writing process. However, the context of the present study differs substantially. The students in this research do not write in English but rather in Spanish as a second language, with Quechua as their mother tongue. This adaptation process is essential because writing in Spanish within the Peruvian school context involves discourse practices and pedagogical approaches that differ from those found in academic English. The present study aims to contribute to the development of culturally relevant assessment tools that more accurately capture beliefs about writing self-efficacy in bilingual contexts. Such tools can guide pedagogical strategies to promote educational equity and the comprehensive development of these students.

In this framework, the objective of the current study was to adapt and validate a Spanish version of the writing self-efficacy scale developed by Sun et al. (2022) for Quechua-speaking students in basic education in Peru.

## 2. Methods

The study was instrumental in nature (Ato et al., 2013), with a cross-sectional design, aimed at adapting and validating a measure of writing self-efficacy for a Quechua–Spanish bilingual population. Data collection was conducted in three public schools belonging to the same *Unidad de Gestión Educativa Local* (UGEL, equivalent to a school district) located in the Peruvian highlands. All schools followed the *National Curriculum for Basic Education* (Ministerio de Educación del Perú, 2016). Spanish was the language of schooling and assessment, while Quechua was the predominant mother tongue among the students. A non-probabilistic convenience sampling method was used. The inclusion criteria were: (a) current enrollment in secondary education, (b) schooling in Spanish as a second language and as the primary language of instruction, (c) informed consent from a parent or legal guardian and student assent, and (d) having Quechua as the mother tongue. The sample size was estimated using Soper’s calculator (2020), considering the number of observed and latent variables in the proposed model, the expected effect size (λ = 0.30), the significance level (α = 0.05), and the desired statistical power (1 − β = 0.80). Although a minimum of 241 participants was calculated, the final sample included 265 students from 1st to 5th year of secondary school, representing various sections within the same UGEL. All participants were residents of the Andean region and native Quechua speakers. The gender distribution was balanced (50.6% female), and most students lived in rural highland areas (97.4%). The largest proportion of students were in the second year of secondary school (35.5%). Regarding writing preferences, 63.8% reported enjoying writing the most when completing school-related assignments. In terms of reading frequency, the most common response was “sometimes” (54.7%). Concerning the educational level of the father or guardian, the most frequent category was “secondary education” (44.2%) (Table 1).

### 2.1. Instruments

*Writing Self-Efficacy*: The Questionnaire of English Writing Self-Efficacy (QEWSE) was used, an instrument designed to assess self-efficacy in English writing (Sun et al., 2022). This questionnaire uses a seven-point Likert scale ranging from 1 (I cannot do it at all) to 7 (I can do it well). The QEWSE was developed by adapting two previous instruments, making specific adjustments to improve the relevance and clarity of certain items. In terms of reliability, the instrument demonstrated high internal consistency, with a Cronbach’s alpha coefficient of 0.95 for the overall scale. At the dimensional level, the coefficients were as follows: ideation (0.70), skills (0.89), written English usage (0.88), and self-regulation (0.78).

Given that, in our context, writing tasks are evaluated in Spanish (second language) and the students’ first language is Quechua, the adaptation process was conceived as a transcultural process and followed international guidelines (Beaton et al., 2000). The adaptation was carried out in four steps:

1. Initial Translation: Two native Spanish-speaking bilingual translators independently translated the QEWSE from English into Spanish. The two versions were then compared and consolidated into a single consensus version.
2. Back-Translation: The Spanish version was translated back into English by two native English speakers from the United States, both fluent in Spanish but without prior knowledge of the original QEWSE. The aim of this step was to ensure that the original meaning of the items remained intact.
3. Expert Committee Review: A committee consisting of two regular basic education teachers experienced in Quechua–Spanish bilingual education and educational research, along with a bilingual psychologist (Quechua–Spanish) trained in psychometrics, reviewed both the Spanish version and the back-translated English versions. Based on this analysis, a preliminary Spanish version of the instrument was developed.
4. Pilot Testing: The preliminary version was administered to a focus group of 15 bilingual students whose first language was Quechua and second language and school language was Spanish. These students shared similar characteristics to the target population. The purpose of this phase was to evaluate comprehension, readability, and cultural relevance of the items. During the pilot test, no terms, expressions, or concepts were identified as confusing or ambiguous by the participants. All items were well understood and answered without difficulty, so no further modifications were made to the preliminary version.

As a result, the Spanish version was confirmed to be clear and appropriate for the target population. It was finalized as the Questionnaire of Spanish Writing Self-Efficacy (QEWS-S) This final version (Table 2) retained the original four-domain structure: ideation, conventions/skills, Spanish writing usage, and self-regulation.

### 2.2. Procedure

The present study was conducted in accordance with ethical principles and received approval from the Ethics Committee of a Peruvian university. Since the sample consisted of secondary school students, informed consent was obtained from parents or legal guardians, along with the students’ voluntary assent prior to their participation. Privacy, confidentiality, and anonymity of the data were strictly ensured, and participation was entirely voluntary, with no academic consequences for students who chose not to participate. The administration of the questionnaire was carried out in person and in group settings within classrooms at the three participating schools, all located in the Andean region of Peru. These schools implement the National Curriculum for Basic Education, where Spanish serves as the language of schooling and assessment, while Quechua is the predominant mother tongue among students. This context ensured that the writing tasks evaluated corresponded to the language in which students engage in formal learning, even though it is not their native language. The administration process was overseen by three trained bilingual (Quechua–Spanish) teachers under the supervision of a general coordinator. The team received standardized training lasting four hours, covering ethical procedures, administration instructions, strategies for addressing participants’ questions, and methods for providing minimal support in Quechua when necessary, without altering the content of the items. Evaluations were conducted during regular school hours, between 8:00 and 11:30 a.m., in quiet, well-lit environments. Students remained in their regular classrooms throughout the assessment process.

### 2.3. Data Analysis

An initial descriptive analysis of the QEWS-S items was conducted, including mean, standard deviation, skewness, and kurtosis, as well as corrected item-total correlations. Skewness (g_1_) and kurtosis (g_2_) values were deemed acceptable within the ±1.5 range (George & Mallery, 2003). Additionally, corrected item-total correlation analysis was applied to identify and exclude items with r(i-tc) ≤ 0.3 or multicollinearity issues (Kline, 2016).

A confirmatory factor analysis (CFA) was subsequently performed to test the scale’s structure, using the MLR estimation method, which is recommended for data that do not meet normality assumptions (Muthen & Muthen, 2017). Model fit was evaluated using several indices: chi-square (χ^2^), the Comparative Fit Index (CFI), and the Tucker–Lewis Index (TLI), with recommended thresholds of ≥0.95; and the Root Mean Square Error of Approximation (RMSEA) and the Standardized Root Mean Square Residual (SRMR), with acceptable values ≤ 0.08 (Kline, 2016; Schumacker & Lomax, 2016).

A second-order CFA was also conducted. In the initial model, the magnitude of factor loadings (λ) was considered acceptable when greater than 0.70 (Dominguez-Lara, 2018). To evaluate internal validity evidence, the Average Variance Extracted (AVE > 0.50) was calculated as an indicator of convergent validity. Discriminant validity was assessed by comparing the AVE with the squared interfactor correlations (AVE > ϕ^2^), following (Fornell & Larcker, 1981).

For reliability estimation, Cronbach’s alpha (α), McDonald’s omega (ω), and the H coefficient were used to assess internal consistency, with values above 0.70 considered acceptable (Hunsley & Marsh, 2008; Ponterotto & Charter, 2009). Scale reliability was established with Cronbach’s alpha and McDonald’s omega coefficients exceeding 0.70, indicating adequate internal consistency (McDonald, 1999).

All statistical analyses were conducted using RStudio Version 2025.09.1-401 (RStudio Team, 2018) with R version 4.1.1 (R Foundation for Statistical Computing, Vienna, Austria; http://www.R-project.org accessed on 15 January 2025). CFA and structural equation modeling were performed using the “lavaan” package (Rosseel, 2012) and measurement invariance analysis was conducted using the “semTools” package (Jorgensen et al., 2012).

## 3. Results

### 3.1. Descriptive Item Statistics

As shown in the table, item means ranged from 4.99 (Item 19) to 5.84 (Item 4); Item 4 received the highest average rating, while Item 19 had the lowest. Regarding skewness (g_1_ and kurtosis (g_2_), most items fell within a moderate range, although some (e.g., Item 4 and Item 23) slightly exceeded a kurtosis value of 2. Several authors consider skewness and kurtosis values between ±2 or ±3 acceptable for most applications in social sciences (George & Mallery, 2003; Kline, 2016). Therefore, although slight deviations from normality were observed, they do not appear severe enough to compromise subsequent analyses. Concerning corrected item-total correlations (r.cor), most values exceeded 0.30, ranging from 0.26 (Item 4) to 0.77 (Item 21). Item 4 had the lowest correlation, though it remained within an acceptable range. The final column, which displays Cronbach’s alpha if each item were deleted, remained around 0.95, indicating that removing any item would not substantially increase internal consistency. Overall, the total Cronbach’s alpha of approximately 0.95 suggests high internal consistency, consistent with the measurement of a robust unidimensional construct (Table 3).

### 3.2. Confirmatory Factor Analysis and Reliability

**Figure 1:**
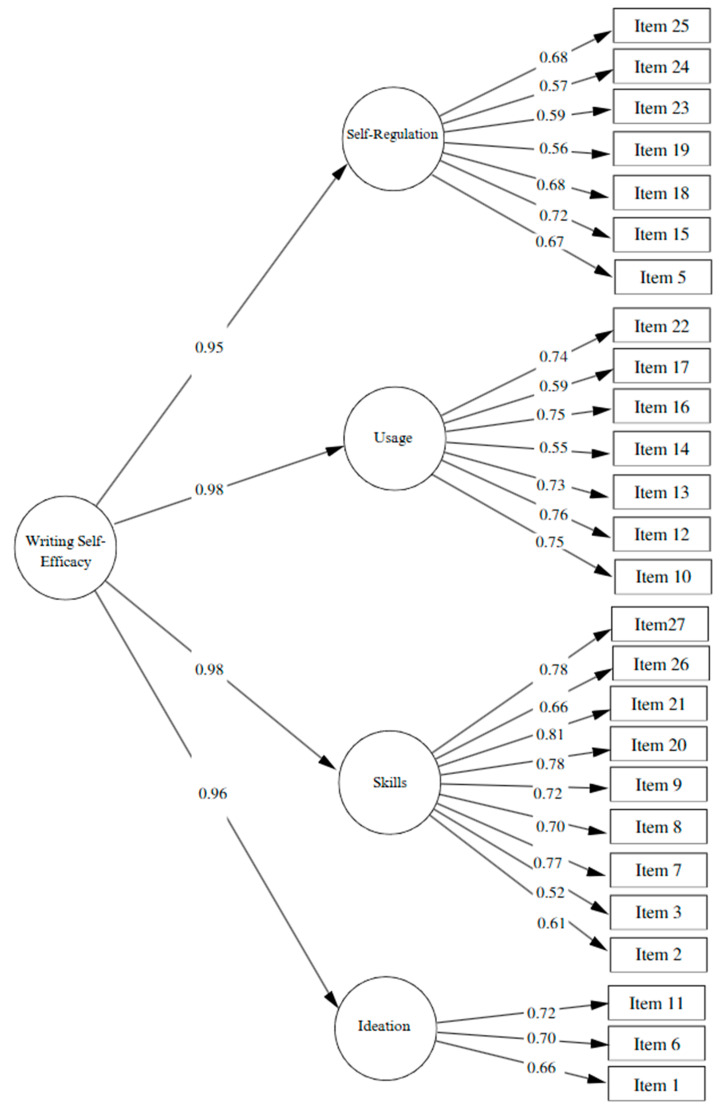
Second-order model.

The factorial structure of the QEWS-S was evaluated through Confirmatory Factor Analysis (CFA) by comparing two models: (a) a first-order four-factor model (Ideation, Skills, Usage, and Self-Regulation) and (b) a second-order hierarchical model, in which these factors were grouped under a general construct of writing self-efficacy (Figure 1). The first-order model demonstrated adequate fit indices: χ^2^ = 545.85, df = 318, *p* < 0.001, CFI = 0.92, TLI = 0.91, RMSEA = 0.05 (90% CI: 0.05–0.06), and SRMR = 0.05. Most factor loadings were satisfactory (λ > 0.50); however, item 4 showed a low loading (λ = 0.26) and was therefore excluded because it compromised the convergent validity of the “Usage” dimension and did not contribute conceptually to the construct. Regarding internal consistency, both Cronbach’s alpha (α) and McDonald’s omega (ω) values were above 0.70 across all dimensions, indicating good reliability. For convergent validity, assessed through the Average Variance Extracted (AVE), the Skills dimension reached a value of 0.51, reflecting adequate convergent validity. In contrast, Ideation and Usage presented values of 0.48, while Self-Regulation reached 0.40, indicating weak convergent validity. AVE values below 0.50 suggest that the items within a dimension do not share sufficient common variance and, therefore, do not optimally explain the underlying construct. With regard to discriminant validity, most AVE values exceeded the shared variances between factors, supporting some degree of conceptual independence. However, high inter-factor correlations were observed (some exceeding 0.90), suggesting substantial conceptual overlap among the evaluated dimensions.

This empirical pattern, together with theoretical foundations that conceptualize writing self-efficacy as an integrated and multidimensional competence, motivated the evaluation of a second-order model (Table 4). This second-order hierarchical model allowed for a more parsimonious representation, where the four specific dimensions (Ideation, Skills, Usage, and Self-Regulation) are derived from a general factor of writing self-efficacy. The second-order model (Figure 1), estimated using robust maximum likelihood (MLR), showed satisfactory fit indices: χ^2^(295) = 517.80 (scaled), CFI = 0.92, TLI = 0.91, RMSEA = 0.06 (90% CI: 0.05–0.07), and SRMR = 0.05. Although the RMSEA indicated a moderate fit, the CFI and TLI values above 0.90 support the appropriateness of the hierarchical structure. Finally, the second-order model demonstrated high overall internal consistency (α = 0.98; ω = 0.98), justifying the reporting of a total writing self-efficacy score in addition to the subscale scores for each specific dimension.

## 4. Discussion

Writing self-efficacy is a fundamental construct in educational research, grounded in Bandura’s social cognitive theory, which posits that individuals’ beliefs about their own abilities directly influence motivation, effort, and academic performance (Bandura, 1997). Previous studies have shown that greater confidence in writing skills is associated with higher quality in produced texts and greater persistence when facing academic challenges (Pajares & Valiante, 1997; Shell et al., 1989). In recent years, there has been increasing emphasis on the importance of adapting self-efficacy measurement instruments to specific cultural and linguistic contexts, particularly in bilingual or Indigenous populations where the language of schooling differs from the students’ mother tongue. In the case of Quechua-speaking students in Peru, Spanish serves as the language of instruction, while Quechua is the mother tongue. This linguistic dynamic can create barriers in both the acquisition and assessment of writing skills, affecting learning outcomes and students’ perception of their competence. However, most available instruments have been developed in Western, urban contexts, limiting their validity and applicability to bilingual and rural populations. The lack of validated scales for these contexts hinders the ability to obtain accurate diagnostic data and design culturally relevant pedagogical strategies, ultimately perpetuating educational and social inequalities. Among the existing scales, the measure proposed by Sun et al. (2022) stands out. This instrument evaluates writing self-efficacy through three dimensions: ideation, conventions, and self-regulation. Nevertheless, this scale was originally developed in English within a monolingual context, making its adaptation to Spanish and validation among Quechua-speaking students essential to reflect their realities and ensure the scale’s validity.

In this study, the initial Confirmatory Factor Analysis (CFA) revealed that a four-factor correlated model (comprising Ideation, Skills, Use, and Self-regulation) showed acceptable fit indices and satisfactory factor loadings (≥0.50 for most items), replicating patterns reported in previous work (Pajares & Valiante, 1997; Shell et al., 1989). These findings support the theoretical conceptualization of writing self-efficacy as a set of specific beliefs encompassing idea generation, technical mastery of writing, practical application, and regulation of the writing process. However, very high inter-factor correlations were observed, particularly between the Skills and Use dimensions (≥0.85), suggesting substantial conceptual overlap. This indicates that students may not clearly differentiate between knowledge of writing skills and their confidence in applying them in academic settings. This phenomenon aligns with previous research showing that, among young learners, beliefs about skills and their application tend to merge into a broader perception of competence (Usher & Pajares, 2008). The results of convergent validity, assessed using the Average Variance Extracted (AVE), showed that only the Skills dimension reached an adequate value (0.51). In contrast, Ideation and Use yielded slightly lower values (0.48), and Self-regulation presented the lowest AVE (0.40). These results suggest that, although the dimensions are related to the overall construct, part of the variance explained by the items is insufficient to guarantee their conceptual independence. Regarding discriminant validity, the comparison between AVE values and shared variances revealed considerable covariation among certain factors, reinforcing the hypothesis that these dimensions are not entirely distinct. Based on these findings, a second-order hierarchical model was evaluated, in which the four specific factors were integrated under a single global dimension of writing self-efficacy. This model demonstrated a comparable fit to the correlated factor model but with greater theoretical and empirical parsimony, justifying its selection as the most appropriate representation of the construct. From Bandura’s (1997) perspective, this reflects how specific beliefs about ideation, skills, use, and self-regulation are integrated into a cohesive system of efficacy beliefs. This structure allows for a comprehensive evaluation of writing self-efficacy while preserving the relevance of each domain. In practical terms, the hierarchical model facilitates reporting a global writing self-efficacy score, while also enabling careful interpretation of subscale scores when a more detailed analysis is needed.

The analysis of internal consistency and reliability confirmed the robustness of the QEWS-S. Cronbach’s alpha values (α = 0.74 to 0.90) and McDonald’s omega values (ω = 0.74 to 0.90) were high and comparable to those reported by Sun et al. (2022) in the original English version (α = 0.70 to 0.89; ω = 0.69 to 0.89). These results demonstrate that the scale maintains its psychometric stability even after cultural and linguistic adaptation to Spanish. Interestingly, the Ideation dimension showed a slight improvement in reliability in this study’s sample (α = 0.74, ω = 0.74) compared to the original scale (α = 0.70, ω = 0.69). This may suggest that the Spanish version better captures this construct among bilingual students. Similarly, the Self-regulation dimension also exhibited higher values (α = 0.82, ω = 0.82 vs. α = 0.78, ω = 0.76), possibly reflecting cultural differences in perceptions of personal control over writing processes, as noted in prior studies on self-efficacy in multilingual contexts (Usher & Pajares, 2008).

### 4.1. Implications

The findings of this study have significant implications for educational practice, policy development, and research in bilingual and intercultural contexts. The QEWS-S for Quechua-speaking students provides a psychometrically robust instrument to assess perceived competence in writing in settings where Spanish is the language of schooling and Quechua is the mother tongue. From an applied perspective, the results highlight the importance of using linguistically and culturally adapted instruments in educational assessment. The QEWS-S makes it possible to identify students with low writing self-efficacy, thereby facilitating the implementation of targeted pedagogical strategies. For instance, programs can be designed to strengthen self-regulation skills—such as planning, monitoring, and revising texts—as well as to build confidence in writing production in the language of instruction. Moreover, it is recommended to incorporate explicit teaching strategies, including modeling writing processes, providing constructive feedback, and fostering collaborative activities that promote motivation and persistence. These approaches can help overcome barriers associated with learning in a second language and improve both academic performance and students’ self-perceptions of competence.

In the realm of educational policy, the results underscore the need to promote linguistic inclusion in evaluation and instruction. Standardized assessments conducted exclusively in Spanish without cultural adaptation may underestimate the abilities of students whose initial literacy development occurred in Quechua. Therefore, educational authorities should integrate validated and culturally relevant instruments, such as the QEWS-S, into monitoring and diagnostic processes. Additionally, teacher training programs should emphasize intercultural approaches and differentiated strategies, equipping educators to meet the needs of bilingual students and reduce educational equity gaps. These policies would foster more inclusive teaching practices aligned with the linguistic diversity of the country.

From a theoretical standpoint, the findings support a hierarchical conceptualization of writing self-efficacy, wherein the dimensions of ideation, skills, use, and self-regulation are integrated into a global belief of competence. This structure enables a comprehensive interpretation of writing self-efficacy while maintaining the flexibility to carefully examine specific profiles within each dimension when a more detailed diagnostic analysis is needed.

### 4.2. Limitations

This study has several limitations that should be considered when interpreting the findings. First, its cross-sectional design prevents the establishment of causal relationships between writing self-efficacy and academic performance. Longitudinal research would make it possible to examine how self-efficacy evolves over time and how it influences the development of writing competence. Second, convenience sampling was used, which limits the generalizability of the findings to other regions or populations. Future studies should employ probabilistic sampling to achieve greater representativeness and geographic diversity. Another limitation concerns the context of data collection. The instrument was administered in a group setting within the classroom, where factors such as noise, fatigue, or differences in instructional quality may have influenced responses. Additionally, although it was confirmed that students were able to read the items in Spanish, their reading and comprehension skills were not directly assessed. This omission could have affected how some students interpreted the questionnaire. Very high correlations were observed between certain dimensions, particularly between Skills and Use, indicating substantial conceptual overlap. This finding supports the decision to adopt a second-order hierarchical model in which these dimensions are integrated into a global belief of writing self-efficacy. While the second-order model provides greater parsimony and theoretical coherence, it also highlights the need for further research using alternative models, such as bifactor or Exploratory Structural Equation Modeling (ESEM). These approaches would allow for a more precise analysis of the relationships between subdimensions and help determine whether the observed similarities are due to the integrated nature of writing in the language of instruction or to specific characteristics of the population studied. Moreover, contextual variables such as access to educational resources, socioeconomic status, or quality of instruction were not controlled for, even though these factors could influence writing self-efficacy. Future studies should incorporate these variables using multilevel models to assess their effects at both individual and institutional levels. Finally, the sample was limited to educational institutions within one region of Peru, which restricts the generalizability of the results to other bilingual contexts or populations with different linguistic combinations.

## 5. Conclusions

The present study makes a significant contribution to the field of educational psychometrics and the assessment of competencies in bilingual and intercultural contexts. By adapting the QEWS-S for Quechua-speaking students, this research addresses a critical need for culturally relevant instruments that reflect the reality of populations historically underrepresented in educational research. The findings demonstrate that the QEWS-S possesses strong psychometric properties and a second-order hierarchical structure, in which the dimensions of ideation, skills, use, and self-regulation are integrated under a global belief of writing self-efficacy. This model provides a more parsimonious and theoretically coherent interpretation, acknowledging the natural interdependence of the various components of the writing process in contexts where Spanish serves as a second language. Adopting a hierarchical model allows for reporting an overall writing self-efficacy score, which is useful for diagnostic and research purposes, while the subdimensions can serve as complementary information for more detailed analyses. Furthermore, the instrument’s high reliability and validity support its use in future research and educational interventions aimed at strengthening students’ confidence and performance in written production.

## Figures and Tables

**Table 1:** Sociodemographic characteristics.

Characteristics		*n*	%
Sex	Female	134	50.6
	Male	131	49.4
Grade Level	Fourth Year	15	5.7
	First Year	71	26.8
	Fifth Year	53	20.0
	Second Year	94	35.5
	Third Year	32	12.1
In which activity do you most enjoy writing?	A. When doing my school assignments.	169	63.8
	B. When communicating on social media (Facebook, Instagram, Twitter, WhatsApp)	96	36.2
How often do you practice reading?	1. Never	1	0.4
	2. Almost never	10	3.8
	3. Sometimes	145	54.7
	4. Almost always	85	32.1
	5. Always	24	9.1
Educational Level of Father or Guardian	Primary education	26	9.8
	Secondary education	117	44.2
	No formal education	7	2.6
	Technical higher education	43	16.2
	University higher education	72	27.2

**Table 2:** English–Spanish Translation.

Items	English Version	Spanish Version
1	I can think of many ideas for my writing	Puedo pensar en muchas ideas para escribir
2	I can organize sentences into a paragraph to express an idea	Puedo organizar oraciones en un párrafo para expresar una idea
3	I can correctly spell all the words in the compositions I write	Puedo deletrear correctamente todas las palabras en las composiciones que escribo
4	I can compose messages in English on social media (e.g., WeChat and blogs)?	Puedo escribir mensajes en español en redes sociales (ej. Facebook, Twitter)
5	I can focus on my writing for at least one hour	Puedo concentrarme en mi escritura durante al menos una hora
6	I can put my ideas into writing	Puedo plasmar mis ideas en escritura
7	I can organize different paragraphs into a composition	Puedo organizar diferentes párrafos en una composición
8	I can correctly use verb tenses in English writing	Puedo usar correctamente los tiempos verbales al escribir en español
9	I can make new sentences with given words	Puedo formar nuevas oraciones con palabras dadas
10	I can write an expository paragraph in English	Puedo escribir un párrafo expositivo en español
11	I can think of appropriate words to describe my ideas	Puedo pensar en palabras adecuadas para describir mis ideas
12	I can focus on the main ideas when writing	Puedo centrarme en las ideas principales al escribir
13	I can write an argumentative paragraph in English	Puedo escribir un párrafo argumentativo en español
14	I can write email messages in English	Puedo escribir mensajes de correo electrónico en español
15	I can finish writing assignments on time	Puedo terminar las tareas de escritura a tiempo
16	I can write a descriptive paragraph in English	Puedo escribir un párrafo descriptivo en español
17	I can write diaries in English	Puedo escribir diarios en español
18	I can plan what I want to say before I start writing	Puedo planificar lo que quiero decir antes de comenzar a escribir
19	I can avoid distractions while I write	Puedo evitar distracciones mientras escribo
20	I can write a paragraph in a cohesive way	Puedo escribir un párrafo de manera cohesionada (que tengan sentido juntas)
21	I can write a sentence with proper grammatical structures	Puedo escribir una oración con estructuras gramaticales adecuadas
22	I can write a narrative paragraph in English	Puedo escribir un párrafo narrativo en español
23	I can revise my writing to make it better	Puedo revisar mi escritura para mejorarla
24	I can control my frustration when I write	Puedo controlar mi frustración al escribir
25	I can keep writing even when it’s difficult	Puedo seguir escribiendo incluso cuando es difícil
26	I can fix my grammar errors	Puedo corregir mis errores gramaticales
27	I can write a paragraph in a coherent way	Puedo escribir un párrafo de manera coherente

**Table 3:** Descriptive Statistics.

Items	M	sd	g_1_	g_2_	r.cor	α
1	5.46	1.09	−0.75	0.64	0.63	0.95
2	5.42	1.11	−0.63	−0.2	0.59	0.95
3	5.58	1.09	−0.67	0.16	0.51	0.95
4	5.84	1.2	−1.59	2.86	0.26	0.95
5	5.49	1.16	−0.95	1.01	0.64	0.95
6	5.62	1.07	−1.38	2.68	0.67	0.95
7	5.13	1.1	−0.66	0.19	0.74	0.95
8	5.11	1.15	−0.55	−0.04	0.67	0.95
9	5.57	1.14	−0.89	0.6	0.69	0.95
10	5.48	1.13	−0.85	1.08	0.71	0.95
11	5.69	1.01	−1.08	1.72	0.67	0.95
12	5.52	1.08	−1.17	1.89	0.73	0.95
13	5.46	1.12	−1.15	2.06	0.68	0.95
14	5.48	1.25	−0.94	0.65	0.52	0.95
15	5.52	1.11	−1.21	2.44	0.68	0.95
16	5.55	1.11	−1.13	1.71	0.71	0.95
17	5.53	1.17	−0.92	0.81	0.58	0.95
18	5.7	0.99	−0.67	−0.09	0.65	0.95
19	4.99	1.36	−0.73	0.01	0.53	0.95
20	5.17	1.26	−0.87	0.85	0.74	0.95
21	5.02	1.26	−0.65	0.21	0.77	0.95
22	5.62	1.03	−1.03	1.56	0.7	0.95
23	5.73	1.09	−1.56	4.04	0.55	0.95
24	5.2	1.35	−0.81	0.16	0.53	0.95
25	5.37	1.15	−0.8	0.58	0.65	0.95
26	5.37	1.18	−0.92	0.84	0.63	0.95
27	5.35	1.13	−0.88	0.63	0.76	0.95

**Table 4:** Four-Factor Model.

Items	F1	F2	F3	F4
1	0.67			
6	0.7			
11	0.71			
2		0.6		
3		0.52		
7		0.77		
8		0.7		
9		0.72		
20		0.78		
21		0.81		
26		0.65		
27		0.78		
4			0.26	
10			0.76	
12			0.75	
13			0.74	
14			0.55	
16			0.75	
17			0.59	
22			0.74	
5				0.68
15				0.73
18				0.68
19				0.55
23				0.59
24				0.56
25				0.67
AVE	0.48	0.51	0.48	0.40
F1		0.85	0.83	0.94
F2	0.92		1.02	0.85
F3	0.91	0.97		0.83
F4	1.01	0.92	0.91	
α	0.74	0.9	0.85	0.82
ω	0.74	0.9	0.85	0.82

Note. F1 = Ideation; F2 = Skills; F3 = Usage; F4 = Self-Regulation. α: Cronbach’s alpha; ω = McDonald’s omega; AVE: Average Variance Extracted. Below the diagonal: inter-factor correlations; above the diagonal: shared variance between factors.

## Data Availability

The original contributions presented in this study are included in the article. Further inquiries can be directed to the corresponding authors.
